# Inhaled prostacyclin analogues in COVID-19 associated acute respiratory distress syndrome: scientific rationale

**DOI:** 10.1186/s43044-021-00208-y

**Published:** 2021-09-16

**Authors:** Eka Prasetya Budi Mulia, Kevin Luke

**Affiliations:** 1 Department of Cardiology and Vascular Medicine, Faculty of Medicine, Universitas Airlangga – Dr. Soetomo General Hospital, Jl. Mayjen Prof. Dr. Moestopo No.6-8, Surabaya, 60286 Indonesia; 2 Faculty of Medicine, Universitas Airlangga, Jl. Mayjen Prof. Dr. Moestopo No.6-8, Surabaya, 60286 Indonesia

**Keywords:** ARDS, COVID-19, Epoprostenol, Prostacyclin

## Abstract

**Background:**

COVID-19 associated acute respiratory distress syndrome (CARDS) is a severe form of SARS CoV-2 infection and affects about 15–30% of hospitalized patients with a high mortality rate. Growing research and data suggest several available drugs with appropriate pharmacological effects to treat COVID-19.

**Main body:**

Prostacyclin analogues are regiments for pulmonary artery hypertension. Prostacyclin analogues are expected to be beneficial in treating CARDS based on at least four rationales: (1) inhaled prostacyclin analogues improve oxygenation, *V*/*Q* mismatch, and act as an ARDS therapy alternative; (2) it alleviates direct SARS-CoV-2-related coagulopathy; (3) increases nitric oxide production; and (4) possible anti-inflammatory effect. Prostacyclin analogues are available in oral, intravenous, and inhaled forms. The inhaled form has the advantage over other forms, such as parenteral administration risks. Previously, a meta-analysis demonstrated the beneficial effects of inhaled prostaglandins for ARDS treatment, such as improved PaO2/FiO2 and PaO2 along with reduced pulmonary artery pressure. Currently, two ongoing randomized controlled trials are evaluating inhaled epoprostenol (VPCOVID [NCT04452669]) and iloprost (ILOCOVID [NCT04445246]) for severe COVID-19 patients.

**Conclusions:**

Inhaled prostacyclin could be considered in patients with refractory, life-threatening hypoxia despite standard management.

## Background

COVID-19 caused by SARS-CoV-2 has posed enormous challenges to healthcare systems in the world. Currently, no therapeutic agent has been thoroughly proven against the disease. Growing research and clinical data regarding the virology and pathophysiology of SARS-CoV-2 suggest several reused drugs with appropriate pharmacological effects and therapeutic efficacy in treating patients with COVID-19 [1].

Most patients hospitalized for COVID-19 develop complications of acute respiratory distress syndrome (ARDS) or respiratory failure. ARDS is an acute inflammatory lung injury associated with increased pulmonary vascular permeability and loss of aerated lung tissue, affecting 23% of mechanically ventilated critically ill patients. In-hospital ARDS mortality is estimated to be between 35 and 46%, depending on the severity of ARDS [2]. However, the mortality rate in COVID-19 with ARDS seems to be higher, ranging from 13 to 73% [3].

Prostacyclin is a potent vasodilator of all vascular beds and an endogenous inhibitor of platelet aggregation. The antithrombotic effect results from the activation of intracellular adenylate cyclase and an increase in cyclic adenosine monophosphate (cAMP) in platelets. These agents include epoprostenol, treprostinil, and iloprost [4, 5]. Inhaled prostacyclin analogues have been used for pulmonary vasodilation for vasoreactivity testing, acute cor pulmonale, post-cardiac surgery patients, and those with ARDS [6, 7].

## Main text

### COVID-19 associated acute respiratory distress syndrome

COVID-19 associated acute respiratory distress syndrome (CARDS) is a severe form of SARS CoV-2 infection and affects about 15–30% of hospitalized patients. The early hypothesis stated that cytokine storm is related to CARDS development since the elevation of serum IL-6, IL-1β, and TNF-α was evident. However, a recent comparison prevailed that COVID-19 serum cytokine levels were significantly lower to sepsis and another cytokine release syndrome. Severe COVID-19 pneumonia case definition overlaps with "classic" ARDS. Yet, CARDS is expected to have unique pathophysiological features to “classic” ARDS, namely endothelial barrier disruption followed by intravascular thrombosis, endothelial dysfunction-related hypoxic pulmonary vasoconstriction loss, and exaggerated blood flow to a collapsed lung. A post-mortem examination on CARDS patients revealed a high thrombus burden in pulmonary capillaries, indicating a thrombotic and microangiopathic vasculopathy, compared to “classic” ARDS [8, 9].

Recent multicenter studies demonstrated CARDS had the same lung morphology and respiratory mechanics of "classic" ARDS. Subsequently, CARDS patients with low static respiratory compliance and high D-dimer concentration were associated with a higher mortality rate. It is proposed that widespread pulmonary vascular thrombosis, pulmonary vascular endotheliitis, and elevated D-dimers are unique to COVID-19 patients. This intravascular pathology would increase the dead space and hypoxemia in CARDS patients [2, 10]. Besides, the widespread pulmonary vascular thrombosis could result in pulmonary hypertension (PH) and right ventricular dysfunction (RVD) [11]. Previous meta-analysis demonstrated that PH and RVD are prevalent in COVID-19 patients (19% and 22%, accordingly) and associated with higher mortality, severity, intensive care unit admission, and mechanical ventilation usage [12].

### Mechanism of action prostacyclin analogues in ARDS

Prostacyclin analogues are the most commonly used regimens for pulmonary artery hypertension. It mimics endogenous prostacyclin (PGI_2_) and binds to a G-protein coupled receptor on vascular smooth muscle and platelets surface. Followingly, cyclic adenosine monophosphate (cAMP) is activated and induces pulmonary artery vasodilatation, vascular smooth muscle relaxation, and inhibits platelet aggregation. It also appears to antiproliferative and cytoprotective properties. Available forms of prostacyclin analogues are oral, inhaled, and intravenous [13].

Prostacyclin analogues potentially treat CARDS for at least four rationales. First, inhaled prostacyclin analogues improve oxygenation, V/Q mismatch, and act as an ARDS therapy alternative [13–15]. Although it has not been linked to better patient outcomes and is not commonly recommended, it can be utilized in severe, life-threatening hypoxia that is resistant to standard ARDS care, as observed in COVID-19 [13].

Second, it alleviates direct SARS-CoV-2-related coagulopathy by controlling platelet activity. Platelet aggregation is inhibited by prostacyclin at high concentrations. A routine dose of prostacyclin analogue would support platelet adherence to the damaged vascular wall and involve in vascular repair, together with thrombus formation. Prostacyclin therapy counteracts the prothrombotic effect of endothelin and may reduce the in situ thrombosis observed in PAH patients and perhaps in CARDS patients [13, 16].

Third, prostacyclin elevated nitric oxide production, leading to more antithrombotic and vasodilatation. As with inhaled nitric oxide, prostacyclin's potent endothelial effects, such as preventing vasoconstriction and platelet aggregation, may significantly affect these CARDS. Inhaled prostacyclin has the added advantage of inhaled nitric oxide because it does not necessitate the use of any specific equipment and can be administered directly through a standard ventilator (close circuit) [13].

Finally, prostacyclin and nitric oxide speculatively have essential anti-inflammatory effects, particularly on monocyte/macrophage function, which may benefit COVID-infected patients [13, 17].

### Inhaled versus other routes of administration

Inhaled and oral prostacyclin analogues offer the benefit of avoiding concerns in parenteral administration, such as pain at the infusion site, line discharge, skin and blood infections, and other unfavorable side effects [18]. However, it remains unclear whether such agents that act on the prostacyclin pathway are equally effective whether administered orally or by inhalation. AbuHalimeh et al. [19] presented two cases in which transition from inhaled treprostinil to either oral treprostinil or selexipag, resulting in worsening clinical condition and hemodynamic profile after, subsequently the hemodynamic and clinical profile improved after switched back to inhalation. These divergent responses may reflect either impaired gastrointestinal absorption with lower systemic levels of the drug and/or a preferential (local) action of the inhaled drug specifically on the pulmonary vasculature [19, 20]. The parenteral route of treprostinil administration of (IV, SC) is bioequivalent at a steady-state, while oral treprostinil yields systemic exposure similar to that of parenteral administration with approximately 17% bioavailability. Inhaled treprostinil yields lower systemic concentrations but with delivered locally to the lungs [21].

In general, the side effects of inhaled prostacyclin are flushing, jaw pain, headaches, nausea, vomiting, diarrhea, and dizziness [4]. In different populations, such as heart failure, PH, RVD, or refractory hypoxemia after cardiothoracic surgery, inhaled prostacyclin is considered safe [22, 23]. No side effects were reported in both populations. A recent meta-analysis also stated that no side effects such as bleeding and organ dysfunction were reported in ARDS patients receiving inhaled prostacyclin therapy [15].

Whereas, the potential risks and challenges of inhalation therapy in COVID-19 patients include aerosolization and blockage of bacterial/viral filters used in ventilator circuits, particularly in epoprostenol. Therefore, placement of the filter in the expiratory port of the ventilation circuit during inhalation therapy is necessary to minimize aerosolization into the room. In addition, airborne precautions similar to those for intubation should be taken.

### Clinical evidence of inhaled prostacyclin analogue in ARDS

Clinical experience with inhaled prostacyclin for patients with ARDS suggests that side effects are rare, although published data are limited. Previous Cochrane review by Afshari et al. [15] stated that nebulized iloprost and epoprostenol reduce PaO_2_/FiO_2_ ratio in patients with ARDS. However, their effect on mortality reduction was unknown. Early studies of inhaled iloprost in patients with ARDS and pulmonary hypertension showed improved oxygenation without adverse effects on pulmonary mechanics or systemic hemodynamics [24]. Inhaled epoprostenol may also improve oxygenation in ARDS patients with hypotension as the most common adverse event based on a study by Dunkley et al. [25].

A meta-analysis evaluated the potential of inhaled prostaglandins (including PGI_2_) in ARDS management. The review includes 25 studies consisted of 10 observational studies, 7 case reports/series, 6 non-randomized trials, and 2 randomized controlled trials. Improvements in PaO_2_/FiO_2_ and PaO_2_ were observed along with reduced pulmonary artery pressure in inhaled prostaglandins group. The baseline oxygenation and ARDS etiology did not interfere with the result. Despite high heterogeneity and risk of bias due to mixed study designs, this meta-analysis provides evidence to support inhaled prostaglandins as ARDS treatment [14].

### Clinical evidence and ongoing trials of inhaled prostacyclin analogues in COVID-19

Several retrospective studies have demonstrated the potential benefit of prostacyclin analogues use, alone or in combination, with better clinical outcomes in COVID-19. A retrospective single-center study by DeGrado et al. [26] reported that in individuals with refractory hypoxemia due to COVID-19, inhaled epoprostenol and inhaled nitric oxide did not elicit meaningful increases in oxygenation parameters. However, the baseline characteristics were markedly different and early administration of inhalation might be beneficial. Sonti et al. [27] also reported clinically significant improvement in PaO_2_/FiO_2_ after the initiation of inhaled epoprostenol in 50% of mechanically ventilated patients. Combination use of inhaled epoprostenol and prone positioning in COVID-19 patients with mechanical ventilation with refractory hypoxemia showed improved oxygenation compared with each treatment individually [28].

To the best of the author's knowledge, there are currently no published prospective or randomized trials on inhaled prostacyclin analogues in CARDS; however, several [29, 30] are currently being prepared or are being carried out. A randomized, double-blind controlled trial comparing the effects of inhaled epoprostenol delivered via a breath-actuated delivery system to placebo on oxygen levels and treatment outcomes in mechanically ventilated COVID-19 patients is currently on phase 2 (VPCOVID [NCT04452669]) [29]. Another inhaled prostacyclin analogue, iloprost, was also under investigation in phase 2 of a single-arm clinical trial investigating the use of inhaled iloprost 20 mcg three times daily for five days in suspected or confirmed patients COVID-19 with hypoxemic respiratory failure (ILOCOVID [NCT04445246]) [30]. Evidence regarding the use of inhaled prostacyclin in CARDS is summarized in Table 1.

**Table 1:** Evidence of inhaled prostacyclin analogues for CARDS

References	Study design (sample size)	Regiment	Outcome
Filippini et al. [31]	Case report (1)	Iloprost	Improved SpO_2_, PO_2_/FiO_2_, and HRCT findings
DeGrado et al. [26]	Retrospective observational (38)	Epoprostenol or nitric oxide	No significant improvement in oxygenation metrics
Sonti et al. [27]	Retrospective observational (80)	Epoprostenol	Fifty percent of patients have a clinically significant improvement in PaO2/FiO2 after the initiation of epoprostenol
Li et al. [28]	Retrospective observational (43)	Epoprostenol (some with PP)	The combination of inhaled epoprostenol and PP improved oxygenation compared to epoprostenol or PP individually
Franco et al. [29]	Randomized controlled trial (actual 11)	Epoprostenol	Respiratory and cardiac/circulatory failure, oxygenation, time to extubation, ICU days, and hospital days (ongoing)
Kharma et al. [30]	Randomized controlled trial (estimated 40)	Iloprost	Oxygenation parameters, rates of intubation, ventilation duration, ICU and hospital LOS, rates of proning, ECMO, and mortality (ongoing)

CARDS, COVID-19 associated acute respiratory distress syndrome; ECMO, extracorporeal membrane oxygenation; HRCT, high resolution computed tomography; LOS, length of stay; PO_2_/FiO_2_, partial pressure arterial oxygen/fraction of inspired oxygen; PP, prone position; SpO2, oxygen saturation

## Conclusions

Despite the lack of high-quality evidence, the use of inhaled prostacyclin analogues is rational as an adjunctive treatment for COVID. Inhaled prostacyclin may be considered in patients with refractory, life-threatening hypoxia despite standard management. The potential benefits include enhanced perfusion preferentially to well-ventilated lung regions, reducing pulmonary pressures, antithrombotic properties, and a relatively good safety profile.

## Data Availability

Not applicable.

## Abbreviations

ARDS: Acute respiratory distress syndrome
cAMP: Cyclic adenosine monophosphate
CARDS: COVID-19 associated acute respiratory distress syndrome
COVID-19: Coronavirus disease 2019
PH: Pulmonary hypertension
RVD: Right ventricular dysfunction
